# The m7G-Related Long Noncoding RNA Signature Predicts Prognosis and Indicates Tumour Immune Infiltration in Colon Cancer

**DOI:** 10.3389/fgene.2022.892589

**Published:** 2022-06-29

**Authors:** Li Liu, Yukang Wu, Wenzheng Chen, Yebei Li, Jiahe Yu, Guoyang Zhang, Pengcheng Fu, Liu Huang, Jianbo Xiong, Zhigang Jie

**Affiliations:** ^1^ Department of Gastrointestinal Surgery, The First Affiliated Hospital of Nanchang University, Nanchang, China; > ^2^ Department of Renal Medicine, The Second Affiliated Hospital of Nanchang University, Nanchang, China; ^3^ College of Clinical Medicine, Hainan Vocational University of Science and Technology, Hainan, China

**Keywords:** m7G, lncRNA, colon cancer, bioinformatics, tumour immunity

## Abstract

With high morbidity and mortality, colon cancer (CC) is considered as one of the most often diagnosed cancers around the world. M7G-related lncRNA may provide a regulatory function in the formation of CC, but the principle of regulation is still unclear. The purpose of this research was to establish a novel signature that may be used to predict survival and tumour immunity in CC patients. Data about CC in TCGA was collected for analysis, coexpression analysis and univariate Cox analysis were used to screen prognostic m7G-related lncRNAs. A consensus clustering analysis based on prognostic m7G-related lncRNAs was applied, and a prognosis model based on least absolute shrinkage and selection operator (LASSO) regression analysis was established. Independent prognostic analysis, nomogram, PCA, clinicopathological correlation analysis, TMB, survival analysis, immune correlation analysis, qRT–PCR and clinical therapeutic compound prediction were also applied. 90 prognostic m7G-related lncRNAs were found, GO and KEGG analysis showed that prognostic m7G-related lncRNAs were mainly related to cell transcription and translation. The results of the consensus clustering analysis revealed substantial disparities in survival prognosis and tumour immune infiltration between two clusters. We built a risk model with 21 signature m7G-related lncRNAs, patients in the high-risk group had a considerably poorer prognosis than those in the low-risk group. Independent prognostic analysis confirmed that patients’ prognosis was linked to their tumour stage and risk score. PCA, subgroups with distinct clinicopathological characteristics were studied for survival, multi-index ROC curve, c-index curve, the survival analysis of TMB, and model comparison tested the reliability of risk model. A tumour immunoassay revealed a substantial difference in immune infiltration between high-risk and low-risk individuals. Five chemicals were eliminated, and qRT–PCR indicated that the four lncRNAs were expressed differently. Overall, m7G-related lncRNA is closely related to colon cancer and the 21 signature lncRNAs risk model can efficiently evaluate the prognosis of CC patients, which has a possible positive consequence for the future diagnosis and therapy of CC.

## Introduction

Colon cancer (CC) is one of the most prevalent diagnosed tumours worldwide, with the third-highest incidence of occurrence (10%) among all cancers, trailing only lung cancer (11.4%) and breast cancer (11.7%) (Sung et al., 2021). After lung cancer (18%), CC is the second largest cause of mortality (9.4%) (Sung et al., 2021). Colonoscopy have spread worldwide which increases the rate of early detection of CC, and improvements in treatments have decreased the mortality of CC, but CC is still one of the critical causes of threat to health (Miller et al., 2019). Therefore, it is urgent to explore new risk factors and biomarkers to predict prognosis and develop new therapeutic targets.

Noncoding transcripts of more than 200 nucleotides are known as long noncoding RNAs (lncRNAs) (Wang et al., 2011). In previous studies, lncRNAs that lack open reading frames were thought to be “useless impurities” in coding RNAs. It is now clear that these “useless impurities” serve vital cellular functions both in epigenetic gene-regulatory mechanisms and at the transcriptional and posttranscriptional levels (Chen, 2016). Several studies have suggested that the effects of lncRNAs are crucial to the pathogenesis and development of colon cancer (Bhan et al., 2017; Ni et al., 2020; Chen et al., 2021). Furthermore, Sen et al. indicated that lncRNA FEZF1-AS1 is beneficial to immune escape in colon cancer *via* the mediation of T reg cell differentiation (Hong et al., 2021). The above mentioned studies imply that lncRNAs have a strong link to tumour immunity.

Nevertheless, the mechanisms of regulating the expression of lncRNAs are largely unclear. Recent studies have suggested that there is a close relationship between the expression of lncRNAs and RNA methylation (Fazi and Fatica, 2019; He et al., 2020; Yi et al., 2020). This process plays a crucial role in multitudinous functions of cells, including RNA splicing (Zhao et al., 2014; Xiao et al., 2016), DNA damage repair (Xiang et al., 2017), translation (Wang et al., 2015), mRNA stability (Du et al., 2016; Lee et al., 2020), immunogenicity (Karikó et al., 2005), immune reponse (Li et al., 2017), and oncogenesis (Zhang et al., 2017; Wang et al., 2020). Previous studies have confirmed that the methylation of different RNAs directly or indirectly mediates tumour immunity (Li et al., 2017; Liu et al., 2019). N7-methylguanosine (m7G), the methylation of the seventh N of RNA guanine, is type of RNA methylation. Jieyi’s study showed that m7G promotes lung cancer progression both in mRNA and tRNA (Ma et al., 2021). Additionally, another study indicated that m7G tRNA modification can enhance the translation of oncogenic mRNA and promote the progression of intrahepatic cholangiocarcinoma (Dai et al., 2021). The above mentioned-studies mentioned that m7G is an oncogenic factor, but the expression and biological function of m7G regulating lncRNAs in cancer are unknown.

In this study, we screened the m7G-related lncRNAs found in CC based on the Cancer Genome Atlas (TCGA) database and constructed an m7G-related prognostic lncRNA model. The purpose was to probe the correlation between m7G-related lncRNAs and CC through bioinformatics analysis and a few experiments.

## Materials and Methods

### Data Collection

Genes related to m7G were obtained prior reviews (Tomikawa, 2018) and GSEA websites (http://www.gsea-msigdb.org/gsea/login.jsp) (Damian and Gorfine, 2004), and genesets were selected from the GSEA website were “GOMF_M7G_5_PPPN_DIPHOSPHATASE_ACTIVITY”, “GOMF_RNA_7_METHYLGUANOSINE_CAP_BINDING,” and “GOMF_RNA_CAP_BINDING.” The TCGA database (https://portal.gdc.cancer.gov/repository) was used to acquire RNA sequencing (RNA-seq) data and clinical characteristics (Liu et al., 2018) on 10 January 2022, including 473 tumour datasets and 41 normal datasets. All data were standardized to fragment per kilobase million (FPKM) (Conesa et al., 2016) values.

### Filtering Prognostic m7G-Related lncRNAs

The “limma” (Ritchie et al., 2015), “ggalluvial,” “ggplot2” and “dplyr” packages were utilized to filter m7G-related lncRNAs and draw the Sankey relational chart. |Pearson R| >0.4 and *p* < 0.001 were the criteria for filtering using Pearson’s correlation analysis. The “survival” package was used to select prognostic m7G-related lncRNAs with *p* < 0.05 using univariate Cox regression analysis (van Dijk et al., 2008). To show the results more vividly, the heatmap and forest plot were drawn by the “pheatmap” and “limma” packages. The degree of difference is marked: ∗ if *p* < 0.05, ∗∗ if *p* < 0.01, and ∗∗∗ if *p* < 0.001.

### Gene Ontology and Kyoto Encyclopedia of Genes and Genomes Analysis

For gene ontology (GO) and Kyoto Encyclopedia of Genes and Genomes (KEGG) analyses, prognostic m7G-related lncRNAs were chosen. GO analysis includes three parts: molecular function (MF), biological process (BP), and cellular component (CC). The “org.Hs.eg.db,” “clusterProfiler,” “ggplot2” and “enrichplot” packages were used for GO analysis and KEGG analyses, and a bubble chart was drawn. The criteria were FDR <0.05 or *p* < 0.05.

### Consensus Clustering and Tumour Immune Analysis

For consensus clustering, prognostic m7G-related lncRNAs were chosen (Cristescu et al., 2015), and the “limma,” and “ConsensusClusterPlu” packages (Wilkerson and Hayes, 2010) were used to subdivide all CC data into different groups. Kaplan-Meier curve (Kaplan and Meier, 1958) were used to draw survival disparities between subgroups, and heatmaps were generated to show the correlation between lncRNA expression levels and clinical features. The “survival,” “survminer,” and “pheatmap” packages were used for drawing.

We probed the differences in the expression of immune cells and immune checkpoints among subgroups. The CIBERSORT algorithm was used for immune scoring, and the differences between clusters were evaluated. The packages “limma,” “estimate,” “reshape2,” “ggplot2,” “ggpubr,” and “vioplot” were used for these analyses. The degree of difference was noted: ∗ if *p* < 0.05, ∗∗ if *p* < 0.01, and ∗∗∗ if *p* < 0.001.

### Establishment of the Risk Model

We employed TCGA expression data files and clinical data files to investigate the predictive utility of prognostic m7G-related lncRNAs for clinical prognosis. Least absolute shrinkage and selection operator (LASSO) regression analysis (Bunea et al., 2011) {risk score = Ʃ [Exp (lncRNA) × coef (lncRNA)]} was used to create a predictive signature for m7G-related lncRNAs, the regression coefficient is coef (lncRNA), and the corresponding expression of the included lncRNAs is Exp (lncRNA). According to a 7:3 ratio, all tumour samples were randomly allocated into training and testing groups. According to the median value of the risk of all patients, the samples are sorted into two categories: high-risk and low-risk. The “survival,” “caret,” “glmnet,” “survminer,” and “timeROC” packages were used in this analysis.

After constructing the risk model, we researched the survival risk of the two groups. Survival curves, receiver operating characteristic (ROC) curves (Hanley and McNeil, 1982), heatmaps, and the areas under the time-dependent ROC curves (AUCs) were rendered by the “survival,” “timeROC,” “survminer,” and “pheatmap” packages.

### Independent Prognostic Analysis, Nomogram

We researched all of the tumour samples’ clinical features. In a Cox regression-based univariate and multivariate independent prognostic analysis, the “survival” package was used to determine whether clinical characteristics (age, sex, and TNM stage) and risk score could be utilized as independent prognostic variables. A nomogram (Iasonos et al., 2008) was used to indicate the prognosis of specific individuals. The efficiency of prediction was assessed using a calibration curve. The terms “survival,” “regplot,” and “rms” were used in this process.

### Principal Component Analysis and Prognostic Analysis of Clinical Features

The total gene expression profile, m7G-related genes, m7G-related lncRNAs, and risk model lncRNAs were all subjected to principal component analysis (PCA) to reduce dimensionality, identify the model, and show the high-dimensional data. We used the “limma” and “scatterplot3d” packages to perform this process. Expression levels and survival analysis of different clinicopathological characteristic subgroups were produced using the “ggpubr” and “limma” packages. This analysis is useful in confirming the accuracy of our risk model.

### Tumour Mutational Burden, Model Comparison and Evaluation

Tumour mutational burden (TMB) reflects the frequency of gene mutations in tumour tissue and is assumed to be related to tumour immunity. To display the TMB results of patients in the high-risk and low-risk groups, we analysed the difference and survival of TMB between the high- and low-risk groups in TCGA. The “maftools,” “limma,” “ggpubr,” “survival,” and “survminer” packages were utilized.

We drew multi-index ROC curves and concordance index curves (C-index curve) in all tumour samples to examine the model’s efficacy (including risk, age, gender, and stage). The “dplyr,” “survival,” “rms,” “pec,” “survminer,” and “timeROC” packages were implemented for this analysis. At the same time, we selected two published articles that constructed the lncRNA risk model in colon cancer to compare with our model. We compare the ROC values between these models and draw the relevant AUC curves. The terms “limma,” “survival,” “survminer,” and “timeROC” were utilized for this comparison. Of note, this comparison used the AUC values of all samples and they were not divided into training group samples or testing group samples.

### Tumour Immune Analysis

The CIBERSORT algorithm was utilized in this study to investigate the relationship between the risk score and immune cells/function. The “reshape2,” “ggpubr,” and “limma” packages were applied for immune cell analysis; the “GSEABase,” “limma,” “GSVA,” “reshape2,” and “ggpubr” packages were applied for immune function analysis, and the relevant boxplots and the bar chart were drawn to present the results more clearly. According to the median of the levels of immune cell enrichment or immune function in all patients, the samples were separated into two groups: those with high immune cell/function and those with low immune cell/function. We analysed the survival differences between the high and low cell/function groups. The “survminer,” “survival,” and “limma” packages were implemented. The degree of difference was noted: ∗ if *p* < 0.05, ∗∗ if *p* < 0.01, and ∗∗∗ if *p* < 0.001.

### Prediction of Potential Therapeutic Compounds

We evaluated the IC50 values of the compounds retrieved from the GDSC website (https://www.cancerrxgene.org/) to predict probable compounds that may be employed in CC treatment. The “pRRophetic,” “limma,” “ggpubr,” and “ggplot2” packages were used to predict the chemicals that may be employed for CC treatment.

### Quantitative Real-Time Polymerase Chain Reaction

The normal human colonic epithelial cell line NCM460, as well as the human CC cell lines HT29 and SW620, were supplied by the Chinese Academy of Sciences’ Shanghai Cell Bank. The TransZol Up Plus RNA Kit was used to extract and purify total RNA (Transgen, Beijing, PRC). For reverse transcription, the EasyScript One-Step gDNA Removal and cDNA Synthesis SuperMix (Transgen, Beijing, PRC) and T100 Thermal Cycler (BIO-RAD, United States) were employed. Quantitative real-time polymerase chain reaction (qRT–PCR) was performed using PerfectStart Green qPCR SuperMix (Transgen, Beijing, PRC) and CFX Connect Optics Module (BIO-RAD, United States). All experimental procedures were carried out following the product manual’s instructions. Considering that the expression of many risk lncRNAs was very low, we chose four lncRNAs that were relatively easy to obtain results for qRT–PCR verification.

For PCR amplification, the primer sequences were as follows:

NIFK-AS1, forward: 5′-TTG​GGT​CTT​CGA​AAG​TGC​TG-3′,

reverse: 5′-ACG​CTC​CAA​AAC​ACT​TTC​CG-3’;

RNF216P1, forward: 5′-GGC​CAG​CCA​AGA​TGA​GAC​AA-3′,

reverse: 5′-TCA​GCA​GCT​TGG​ATG​AAG​CA-3’;

ZEB1-AS1, forward: 5′-GGT​TTC​CTT​CCT​GCT​TCC​CA-3′,

reverse: 5′-ACT​CCG​GTC​ACG​TTT​CAG​TT-3’;

ZKSCAN2-DT, forward: 5′-TCT​GGC​GGA​AGT​ATC​TGT​GC-3′,

reverse: 5′-AGC​ACC​AGA​AGA​GAG​CAA​GC-3’;

GAPDH, forward: 5′-CCC​ACT​CCT​CCA​CCT​TTG​AC-3′,

reverse: 5′-CCA​CCA​CCC​TGT​TGC​TGT​AG-3’;

GAPDH was utilized as an internal control and each sample was reproduced three times. The relative expression levels were determined using the 2-ΔΔCt method. T-tests were used to compare the expression of NIFK-AS1, RNF216PA, ZEB1-AS1, and ZKSCAN2-DT (mean ± SEM). The graphs were made with GraphPad Prism (version 8.0.2). The degree of difference was noted: ∗ if *p* < 0.05, ∗∗ if *p* < 0.01, and ∗∗∗ if *p* < 0.001.

### Statistical Analysis

R software (version 4.1.0) was used for statistical analysis and result visualization. The differential expression was authenticated using the Benjamini–Hochberg technique. The mRNA levels of pyroptosis-related lncRNAs were determined using the Mann–Whitney U test. The differences between the two groups were determined using student’s *t*-test. The chi-square test was used to compare the categorization variables in the training and testing tests. The Pearson correlation test was used to analyse the relationship between subtypes, clinicopathological variables, risk score, immunological check inhibitors, and immune infiltration levels. For survival analysis, the Kaplan–Meier technique was used, along with a two-sided log-rank test.

## Result

### Prognosis-Related lncRNAs With Coexpression of m7G

**FIGURE 1:**
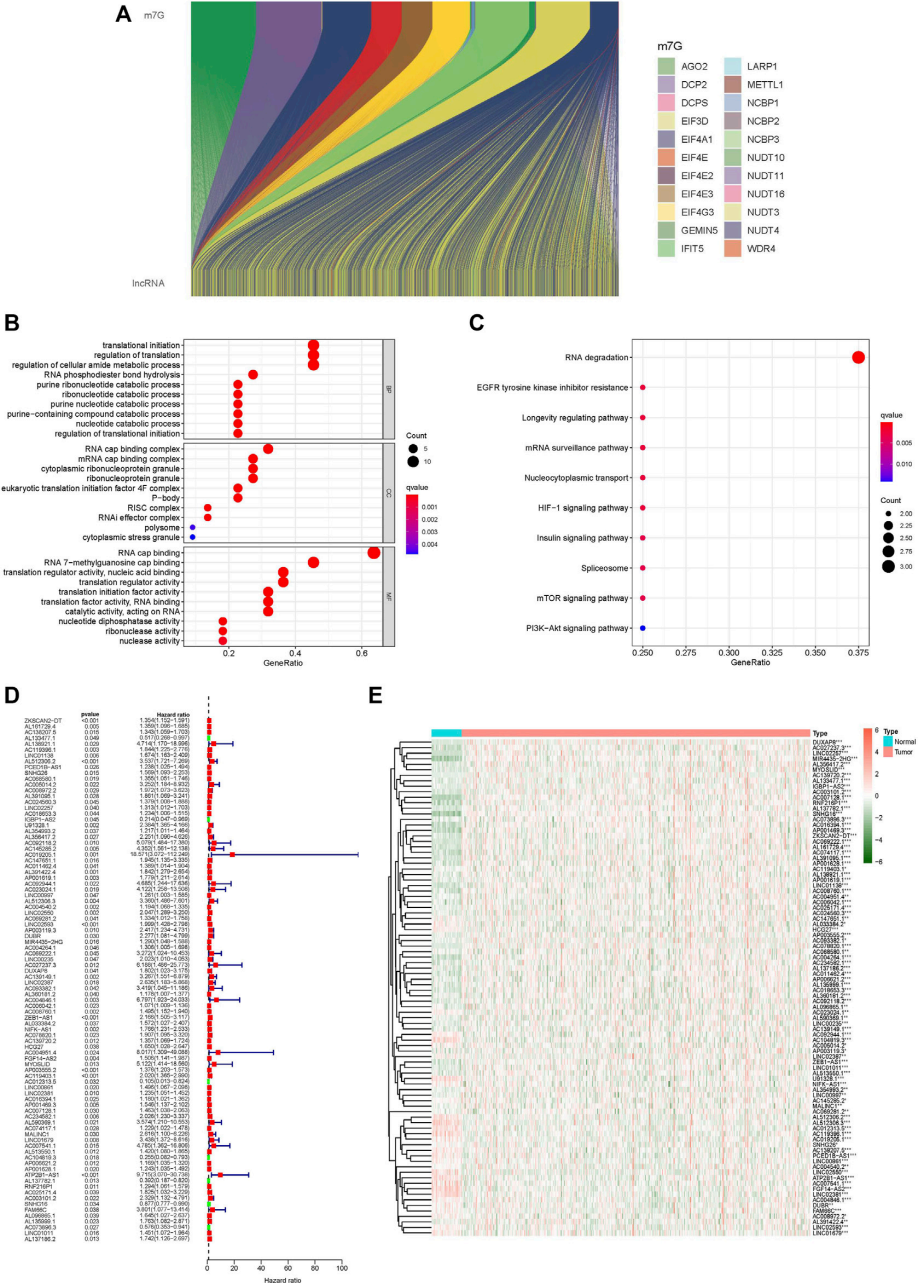
GO and KEGG analysis of m7G-related lncRNAs, differentially expressed lncRNAs. **(A)** Sankey relational diagram for m7G genes and m7G-related lncRNAs, **(B)** GO analysis of m7G-related lncRNAs, **(C)** KEGG analysis of m7G-related lncRNAs, **(D)** Forest plot of differentially expressed m7G-related lncRNAs, **(E)** Heatmap of differentially expressed m7G-related lncRNAs. *p < 0.05, **p < 0.01, ***p < 0.001.

We identified 1,627 lncRNAs in CC that coexpressed m7G-related genes (|Pearson R| > 0.4 and *p* < 0.001) (Figure 1A). Following that, 90 lncRNAs associated with prognosis were uncovered using univariate cox analysis (Figures 1D,E): ZKSCAN2-DT, AL161729.4, AC138207.5, AL133477.1, AL138921.1, AC119396.1, LINC01138, AL512306.2, PCED1B-AS1, SNHG26, AC068580.1, AC005014.2, AC008972.2, AL391095.1, AC024560.3, LINC02257, AC018653.3, IGBP1-AS2, U91328.1, AL354993.2, AL356417.2, AC092118.2, AC145285.2, AC019205.1, AC147651.1, AC011462.4, AL391422.4, AP001619.1, AC092944.1, AC023024.1, LINC00997, AL512306.3, AC004540.2, LINC02550, AC069281.2, LINC02593, AP003119.3, DUBR, MIR4435-2HG, AC004264.1, AC069222.1, LINC00235, AC027237.3, DUXAP8, AC139149.1, LINC02387, AC093382.1, AL360181.2, AC004846.1, AC006042.1, AC008760.1, ZEB1-AS1, AL033384.2, NIFK-AS1, AC078820.1, AC139720.2, HCG27, AC004951.4, FGF14-AS2, MYOSLID, AP003555.2, AC119403.1, AC012313.5, LINC00861, LINC02381, AC016394.1, AP001469.3, AC007128.1, AC234582.1, AL590369.1, AC074117.1, MALINC1, LINC01679, AC007541.1, AL513550.1, AC104819.3, AP006621.2, AP001628.1, ATP2B1-AS1, AL137782.1, RNF216P1, AC025171.4, AC003101.2, SNHG16, FAM66C, AL096865.1, AL135999.1, AC073896.3, LINC01011, and AL137186.2.

### Gene Ontology and Kyoto Encyclopedia of Genes and Genomes Analysis

We performed GO and KEGG analyses of m7G-related lncRNAs. GO analysis included CC and MF of BP, and the significant correlations of lncRNAs were analysed at the cellular constituent, molecular function, and biochemical pathway levels in GO analysis. The obtained results revealed that m7G-related lncRNAs were primarily related to a change in the transcription and translation processes in cellular constituents, the function of m7G and the translation process in molecular function, and protein translation and nucleotide metabolism in biological processes (Figure 1B). KEGG analysis helped us to understand which pathways m7G-related lncRNAs are mostly associated with in colon cancer, and the outcome revealed that m7G-related lncRNAs were primarily associated with RNA regulation (Figure 1C).

### Consensus Cluster Analysis of Prognostic m7G-Related lncRNAs

**FIGURE 2:**
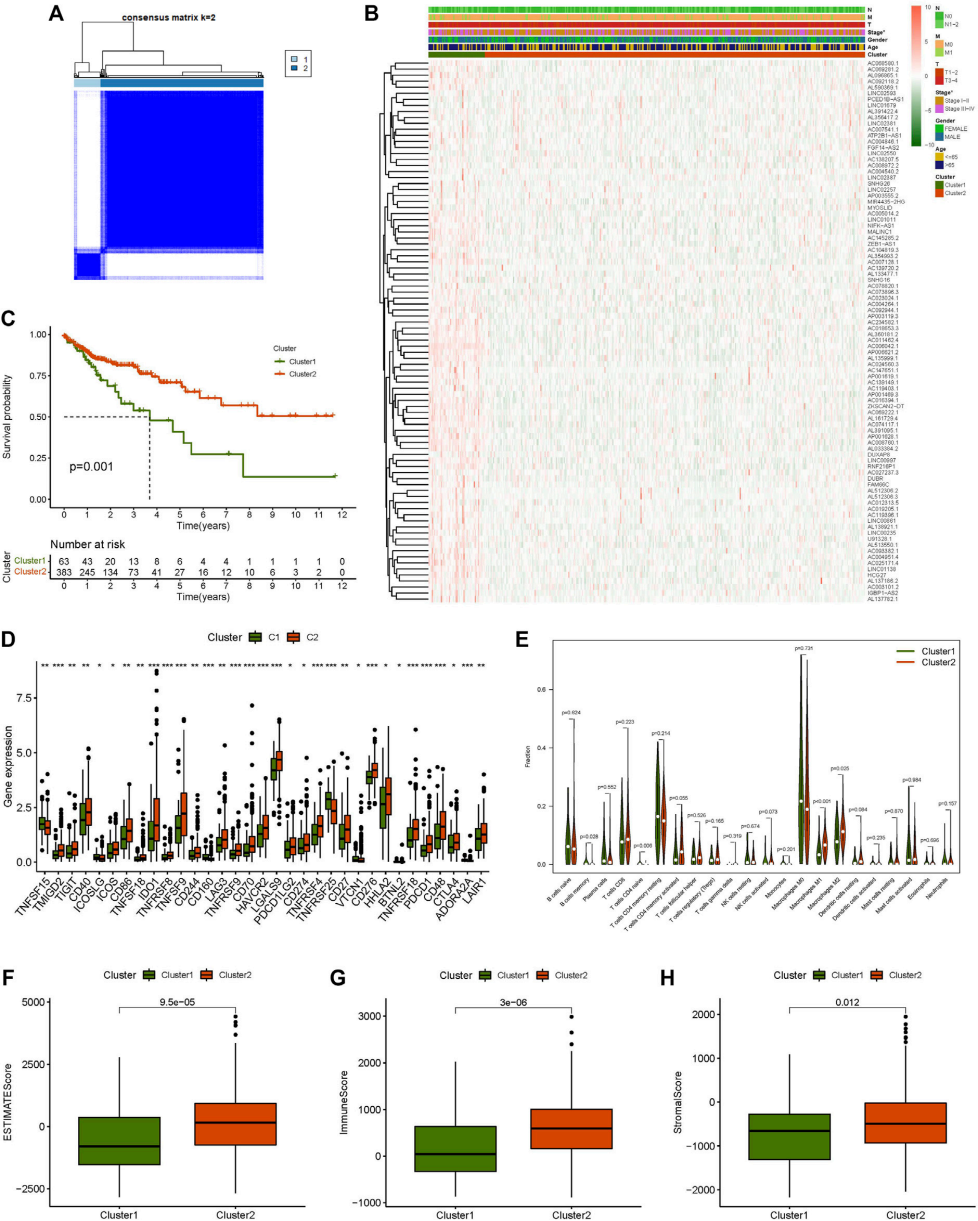
Consensus clustering analysis and immune correlation analysis. **(A)** Consensus clustering matrix for k = 2, **(B)** Heatmap of m7G-related lncRNAs expression and clinicopathologic features in clusters 1 and cluster 2, **(C)** Kaplan-Meier curves of overall survival (OS) in clusters 1 and cluster 2, **(D)** Expression level of immune checkpoint in clusters 1 and cluster 2, **(E)** Relative contents of different immune cells in cluster 1 and cluster 2, **(F)** ESTMATE score in clusters 1 and cluster 2, **(G)** Immune score in clusters 1 and cluster 2, **(H)** Stromal score in clusters 1 and cluster 2. *p < 0.05, **p < 0.01, ***p < 0.001.

The correlation between 90 prognostic m7G-related lncRNA expression levels and subgroups in all CC samples was investigated using consensus cluster analysis. From the figure of increasing the clustering variable (k) from 2 to 9, there were the highest intragroup correlations and the lowest intergroup correlations when the clustering variable (k) was set to 2. (Figure 2A; Supplementary Figures S2A–C). The survival analysis revealed a substantial disparity in survival probability between Clusters 1 and 2, with patients in Cluster 2 having a better prognosis than those in Cluster 1. (Figure 2C). The expression levels and clinicopathological heatmap between the two clusters revealed that the tumour stage differed markedly between the two clusters (Figure 2B). The obtained results revealed that there were significant disparities between the two subgroups by analysing the difference in the expression levels of immune checkpoints (including C10orf54, CD200, TNFSF18, CD160, ADORA2A, TNFRSF25, CD28, LGALS9, CD244, TNFSF14, CD40, IDO2, TNFSF15, LAIR1, CD86, ICOSLG, CD70, BTNL2, PDCD1LG2, ICOS, IDO1, TNFSF4, CTLA4, NRP1, LAG3, TNFRSF9, TNFRSF18, CD80, TIGIT, TNFSF9, KIR3DL1, CD200R1, PDCD1, TNFRSF8, TNFRSF14, CD276, TNFRSF4, HHLA2, CD274, CD27, HAVCR2, BTLA, TMIGD2, CD48, CD40LG, VTCN1, and CD44) (Figure 2D). The results of multiple analyses of immune cell content indicated that there were remarkable differences in the content of M1 macrophages, M2 macrophages, and naive CD4 T cells between the two subgroups (Figure 2E). Simultaneously, there was a statistically significant difference in immunological score between the two subgroups; Cluster 2 had a significantly higher score (ESTMATE score, stromal score, and immune score) than Cluster 1 (Figures 2F–H). These findings suggest that m7G-related lncRNAs have a substantial connection with tumour immunity in CC, implying that m7G-related lncRNAs would provide a novel reference marker for CC immunotherapy.

### Establishment of Risk Model

All tumour samples were randomly divided into training and testing groups in a 7:3 ratio. We established a LASSO regression model and selected 21 lncRNAs (including ZKSCAN2-DT, AL512306.2, AC005014.2, U91328.1, AL354993.2, AC092944.1, LINC00997, AC004540.2, LINC02593, AC004846.1, ZEB1-AS1, AC078820.1, AC139720.2, AP003555.2, AC012313.5, AL513550.1, AC104819.3, AL137782.1, RNF216P1, AC003101.2, and AC073896.3) as signature factors from 90 prognostic m7G-related lncRNAs (Supplementary Figures S2D, E). The following formula was used to obtain the risk score: risk score = (0.176943613 ∗ ZKSCAN2-DT exp.) + (0.933836642 ∗ AL512306.2 exp.) + (0.326054032 ∗ AC005014.2 exp.) + (0.111829011 *U91328.1 exp.) + (0.064972028 ∗ AL354993.2 exp.) + (1.771428152 ∗ AC092944.1 exp.) + (0.105833265 ∗ LINC00997 exp.) + (0.041148055 ∗ AC004540.2 exp.) + (0.309526196 ∗ LINC02593 exp.) + (0.771493133 ∗ AC004846.1 exp.) + (0.027471189 ∗ ZEB1-AS1 exp.) + (0.430076387 ∗ AC078820.1 exp.) + (0.162469055 ∗ AC139720.2 exp.) + (0.225983414 ∗ AP003555.2 exp.) + (−2.028167065 ∗ AC012313.5 exp.) + (0.255834323 ∗ AL513550.1 exp.) + (−0.568872124 ∗ AC104819.3 exp.) + (−0.402855251 ∗ AL137782.1 exp.) + (0.122413149 ∗ RNF216P1 exp.) + (0.47174027 ∗ AC003101.2 exp.) + (−0.247170433 ∗ AC073896.3 exp.). We classified the risk score as high risk or low risk based on the median risk score. All high-risk samples were defined as high-risk groups, while low risk-score samples were defined as low-risk groups.

**FIGURE 3:**
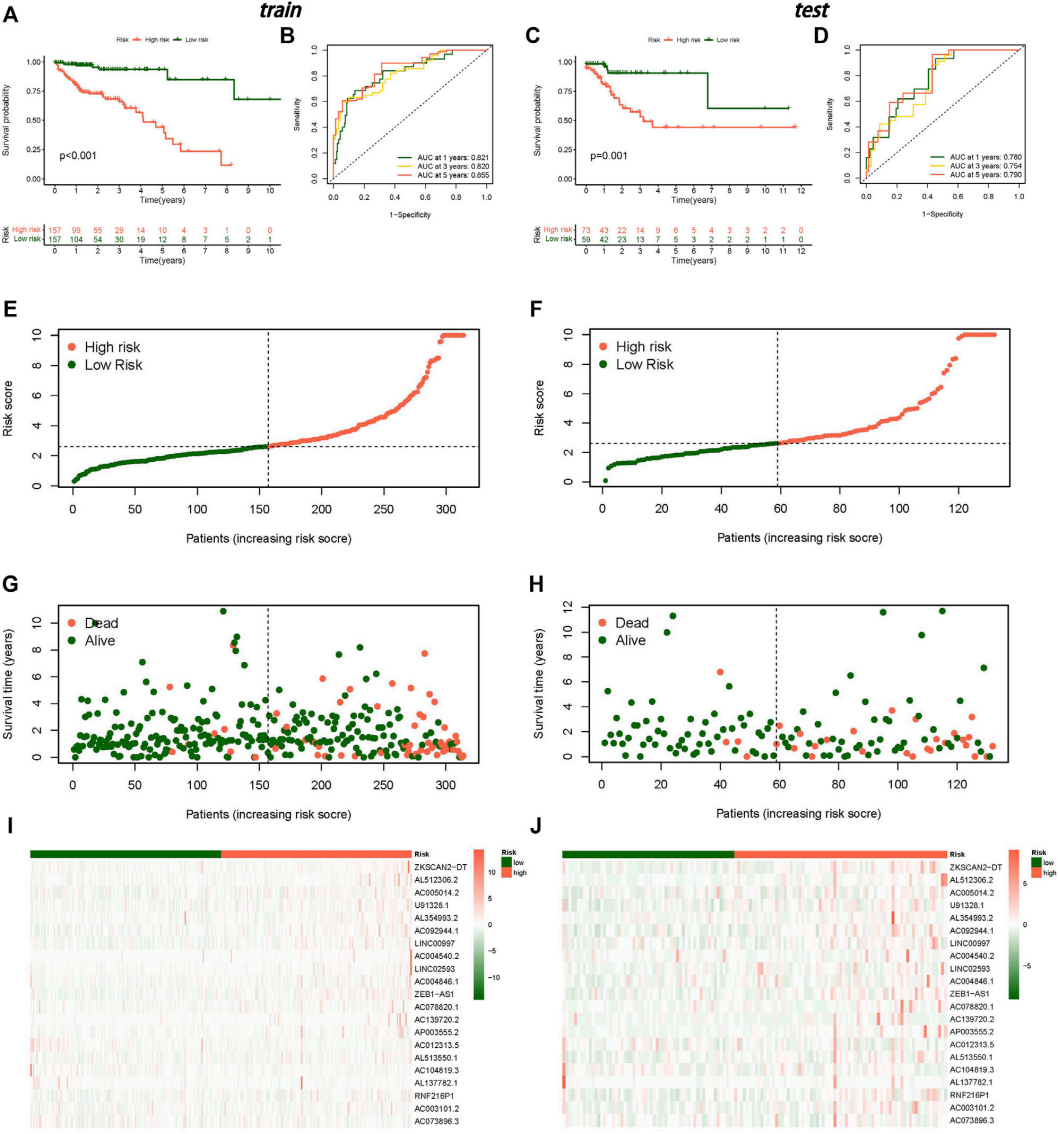
Establishment of the risk model. **(A)** Kaplan-Meier curve for OS in the training group, **(B)** ROC curve in the training group, **(C)** Kaplan-Meier curve for OS in the testing group, **(D)** ROC curve in the testing group, **(E)** Risk score distribution in the training group, **(F)** Risk score distribution in the testing group, **(G)** OS statu in the training group, **(H)** OS statu in the testing group, **(I)** Heatmap of risk lncRNAs expression in the training group, **(J)** Heatmap of risk lncRNAs expression in the testing group.

**FIGURE 4:**
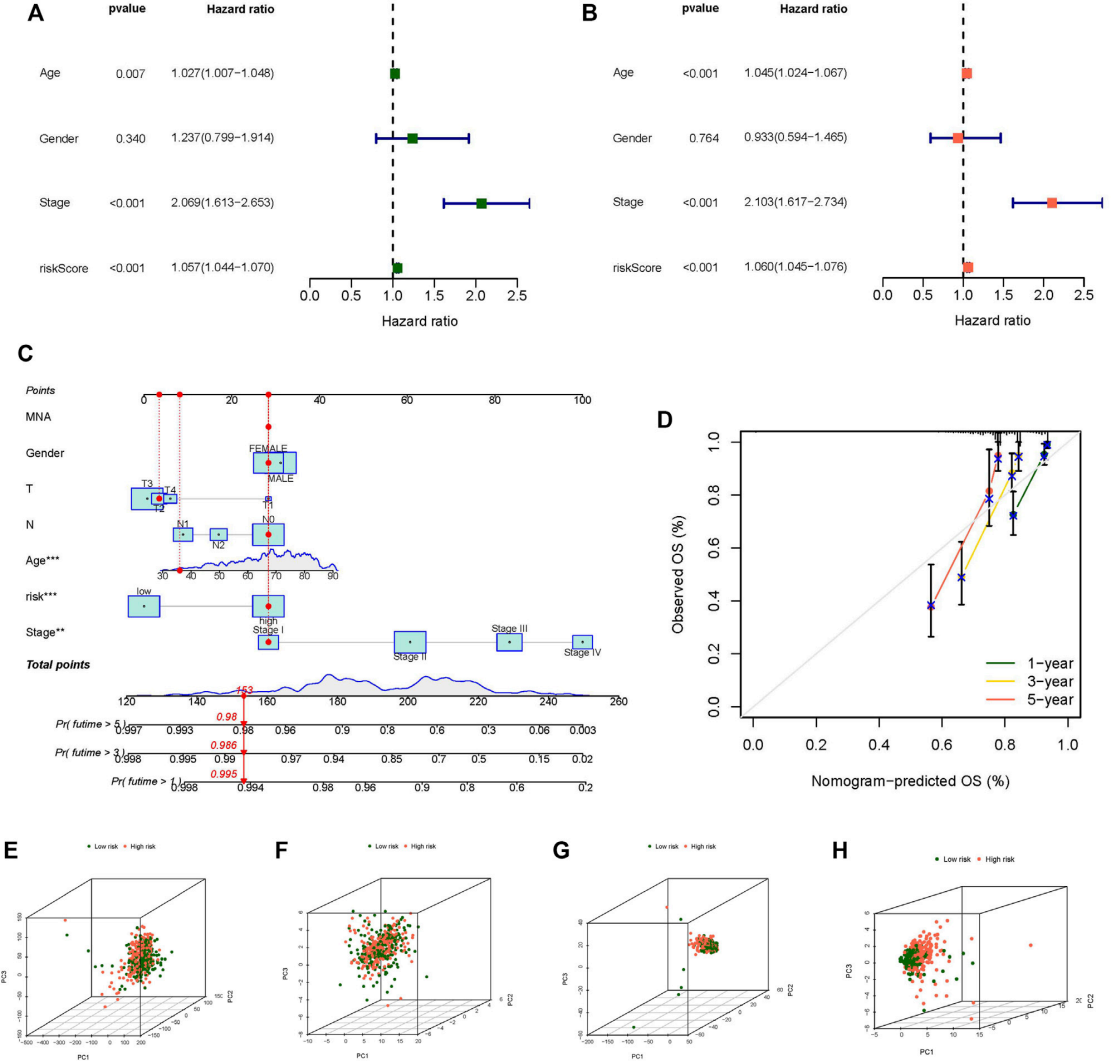
Independent prognostic analysis, Nomogram and principal component analysis. **(A)** Univariate independent prognostic analysis in all tumor samples, **(B)** Univariate independent prognostic analysis in all tumor samples, **(C)** The nomogram predicts the probability of the 1-, 3-, and 5-years OS, **(D)** The calibration plot of the nomogram predicts the probability of the 1-, 3-, and 5-years OS, **(E)** PCA of all genes, **(F)** PCA of m7G genes, **(G)** PCA of m7G-related lncRNAs, **(H)** PCA of risk lncRNAs. *p < 0.05, **p < 0.01, ***p < 0.001.

Regardless of whether the patients were in the training or testing groups, the survival analysis revealed that the survival probability of patients in the low-risk group was greater than that of patients in the high-risk group, and there was a statistically significant difference. (Figures 3A,C). The AUCs of the training and testing cohorts were 0.821 and 0.780 after 1 year, 0.820 and 0.754 at 3 years, and 0.855 and 0.790 after 5 years, respectively (Figures 3B,D), which indicates that the risk model performed well in prediction. The risk curve demonstrated that the death rate of patients rose with the rise of the risk score in both the training and testing groups (Figures 3E–H). The heatmap revealed a significant variation in the expression levels of 21 lncRNAs between the high-risk and low-risk groups (Figures 3I,J). The outcomes of univariate and multivariate independent prognostic analyses revealed that the stage and risk score may be utilized as independent prognostic factors to assess the prognosis of CC patients (Figures 4A,B). These analyses indicated that our risk model has a strong capacity to predict patient prognosis and may provied new ideas for the diagnosis and therapy of CC in the future.

### Nomogram and PCA Verification

We constructed a nomogram to predict the prognosis of individual patients with CC (Figure 4C), and the calibration plot of the nomogram confirmed that our nomogram has good prediction ability (Figure 4D). We used PCA to evaluate the differences between the low- and high-risk groups in four expression profiles (m7G-related genes, total gene expression profiles, m7G-related lncRNAs, and the risk model classified by the expression profiles of the 21 m7G-related lncRNAs). Compared to the other three expression profiles, the separation between high-risk samples and low-risk samples was more clear in the 21 m7G-related lncRNA expression profile (Figures 4E–H). The findings revealed that 21 m7G-related lncRNAs had the strongest discriminating capacity, distinguishing between low- and high-risk groups rather effectively.

### Model Grouping Verification of Clinicopathological Correlation

**FIGURE 5:**
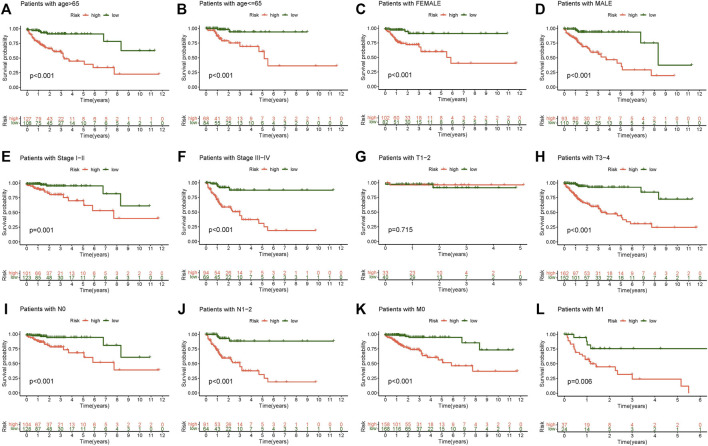
Kaplan-Meier survival subgroup analysis for differential clinicopathological features in high and low-risk scores group. **(A)** Patients with age >65, **(B)** Patients with age ≤65, **(C)** Patients with female, **(D)** Patients with male, **(E)** Patients with stage I-II, **(F)** Patients with stage III-IV, **(G)** Patients with stage T1-2, **(H)** Patients with stage T3-4, **(I)** Patients with stage N0, **(J)** Patients with stage N1-2, **(K)** Patients with stage M0, **(L)** Patients with stage M1.

We discussed the association between the CC risk score and survival. The Kaplan-Meier curve for OS was generated in different clinicopathological characteristic subgroups to demonstrate the difference. Overall, patients in the low-risk group had a considerably greater survival rate than those in the high-risk group (Figure 5). The survival analysis of this clinicopathological characteristic subgroup also proved the effect of the risk score on the prognosis of CC patients and reaffirmed the credibility of the risk model established in our research.

### Analysis of Tumour Mutational Burden

**FIGURE 6:**
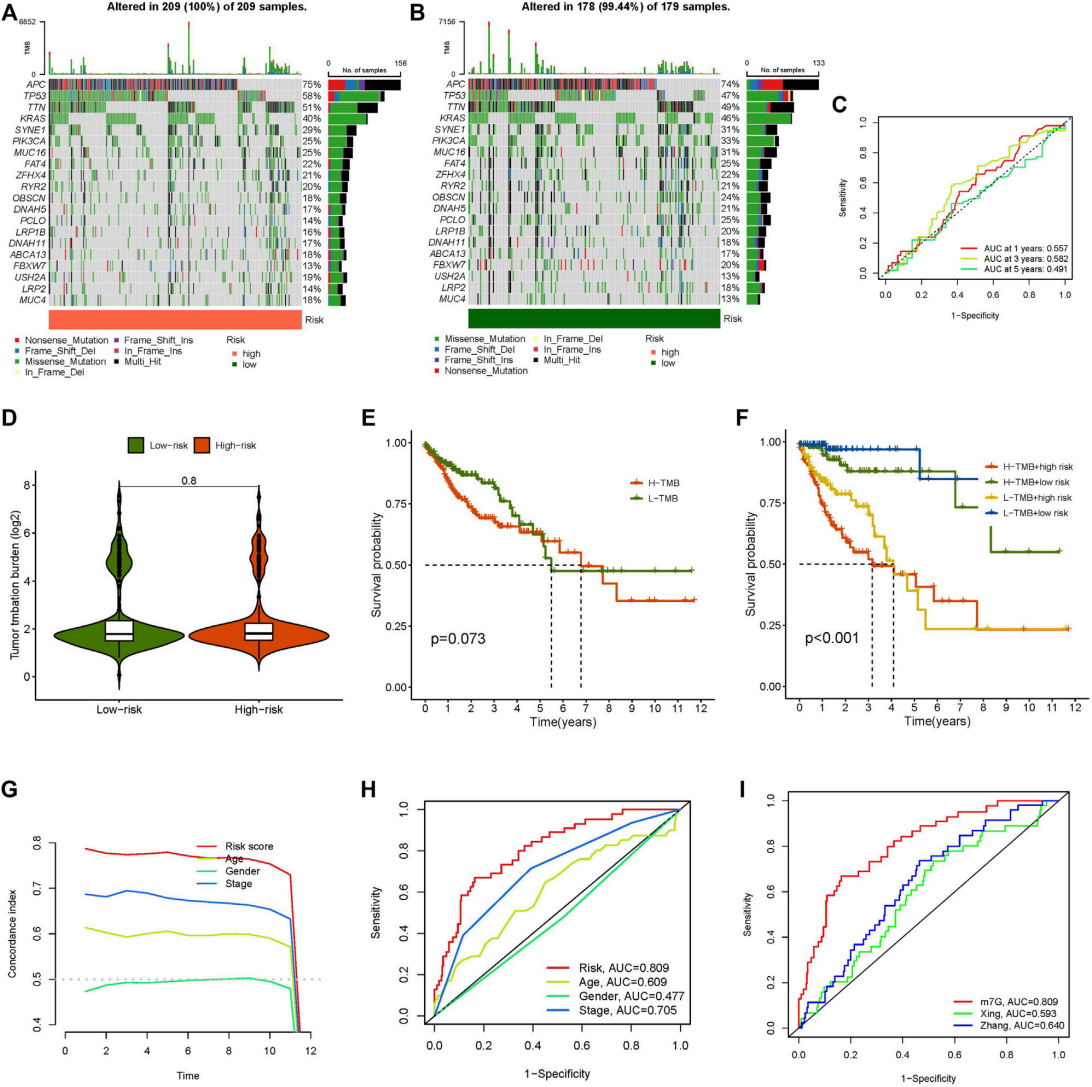
Tumour mutational burden, concordance index curve, multi-index ROC curve and comparison of different lncRNAs models in colon tumors. **(A)** The waterfall plot displays the top 20 genes with high mutation frequency in the high-risk group, **(B)** The waterfall plot displays the top 20 genes with high mutation frequency in the low-risk group, **(C)** ROC curve of TMB score, **(D)** The difference of TMB in high- and low-risk groups, **(E)** Kaplan-Meier curve analysis of OS in high and low TMB score groups, **(F)** Kaplan-Meier curve analysis of OS is shown for patients classified according to the TMB and risk model, **(G)** concordance index curve, **(H)** Multi-index ROC curve, **(I)** ROC curves of different risk models.

TMB represents the mutation rate of genes in the coding area, which is correlated with tumour formation and progression. Based on the TMB score produced from the somatic mutation data of TGCA, our study revealed that the TMB of the high-risk group was greater than that of the low-risk group (Figures 6A,B). The AUCs of the TMB score were 0.557 after 1 year, 0.582 at 3 years, and 0.491 after 5 years (Figure 6C). Compared with the risk score, the AUC value of the TMB score was lower. On the other hand, the difference between the high-risk and low-risk groups was extremely minor, and there was no significant variation in TMB between the two groups (Figure 6D). According to the TMB score, we divided all samples into high- and low-mutation groups and conducted a survival analysis on them combined with the grouping of high- and low-risk groups. The results again was confirmed that TMB did not affect the prognosis of patients with CC, but the predictive effect of the risk score was significant (Figures 6E,F). These results showed that the model’s prediction ability was unquestionably higher than that of TMB.

### Verification of the Credibility of the Risk Model

We created a concordance index curve and a multi-index ROC curve to validate the credibility of the risk model developed in our study. By comparing the AUC values among the various indicators (age, gender, stage, and risk score), we discovered that the AUC value of the risk score was the highest, at 0.809, followed by stage at 0.705, age at 0.609, and gender at 0.477 (Figures 6G,H). We selected two published articles about lncRNA model prediction in colon cancer and compared the AUC value of the published model and our risk model. The outcome indicated that the AUC value of the model researched by Xing (Xing et al., 2021) and Zhang (Zhang et al., 2021) was lower than that in our study (Figure 6I). This result shows that our approach is capable of accurately predicting the prognosis of CC patients.

### Immune Correlation Analysis

**FIGURE 7:**
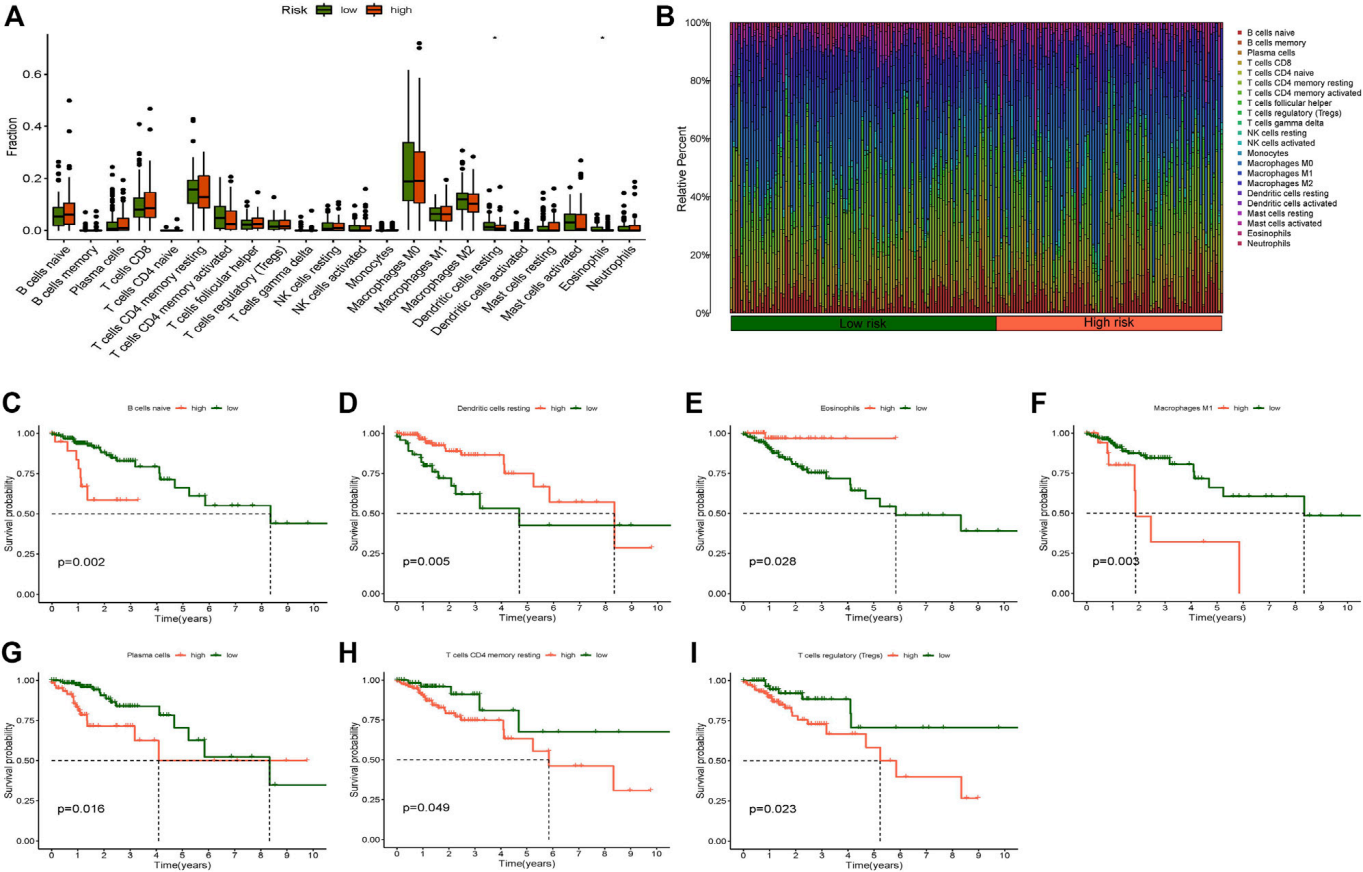
Immune cell infiltration analysis. **(A)** Boxplot of immune cells difference in high- and low-risk group, **(B)** Relative percent of different immune cells in high- and low-risk group, **(C)** Kaplan-Meier survival analysis in naive B cells, **(D)** Kaplan-Meier survival analysis in dendritic cells resting, **(E)** Kaplan-Meier survival analysis in eosinophils, **(F)** Kaplan-Meier survival analysis in macrophages M1, **(G)** Kaplan-Meier survival analysis in plasma cells, **(H)** Kaplan-Meier survival analysis in T cells CD4 memory resting, **(I)** Kaplan-Meier survival analysis in T cells regulatory (Tregs). *p < 0.05, **p < 0.01, ***p < 0.001.

We examined the correlation between the risk model and immune cells/function. The filtration standard was set at *p* < 0.05. The obtained result, where significant difference existed in the proportion of both resting dendritic cells and eosinophils between the two risk groups, indicated that the risk score was clearly correlated with resting dendritic cells and eosinophils (Figure 7A). A bar graph was also generated to display the proportion of each immune cell in the high- and low-risk categories (Figure 7B). The survival analysis of the high and low immune cell groups was researched: resting dendritic cells, naive B cells, M1 macrophages, eosinophils, resting memory T CD4 cells, plasma cells, and regulatory T cells (Tregs) showed significant differences (Figures 7C–I). The prognosis of the low-score group was much better than that of the high-score group in naive B cells, M1 macrophages, plasma cells, T regulatory cells (Tregs), and resting memory CD4 T cells. In resting dendritic cells and eosinophils, the high score group had a higher survival probability than the low score group.

**FIGURE 8:**
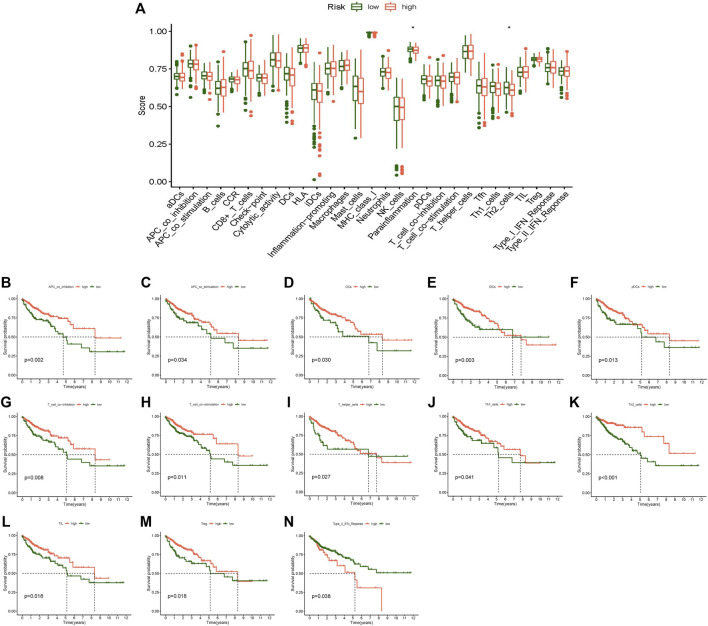
Immune-related function analysis. **(A)** Boxplot of immune-related function score difference in high- and low-risk group, **(B)** Kaplan-Meier survival analysis in APC co-inhibition, **(C)** Kaplan-Meier survival analysis in APC co-stimulation, **(D)** Kaplan-Meier survival analysis in DCs, **(E)** Kaplan-Meier survival analysis in iDCs, **(F)** Kaplan-Meier survival analysis in pDCs, **(G)** Kaplan-Meier survival analysis in T cell co-inhibition, **(H)** Kaplan-Meier survival analysis in T cell co-stimulation, **(I)** Kaplan-Meier survival analysis in T helper cells, **(J)** Kaplan-Meier survival analysis in Th1 cells, **(K)** Kaplan-Meier survival analysis in Th2 cells, **(L)** Kaplan-Meier survival analysis in TIL, **(M)** Kaplan-Meier survival analysis in Treg, **(N)** Kaplan-Meier survival analysis in Type II IFN Reponse. *p < 0.05, **p < 0.01, ***p < 0.001.

In addition, immune function analysis showed that a strong relationship existed between the risk score and immune-related functions of parainflammation and Th2 cells (Figure 8A). Survival examination of several immune functions revealed that APC coinhibition, APC costimulation, DCs, iDCs, pDCs, T-cell coinhibition, T-cell costimulation, T helper cells, Th1 cells, Th2 cells, TILLs, Tregs, and Type II IFN reponse differed significantly between the high- and low-score groups (Figure 8B–N). The low score group had a greater survival rate than the high score group in the Type II IFN reponse function, while other functions were the opposite. In the future, these investigations may serve as a reference for tailored therapy of CC patients.

### Prediction of Potential Therapeutic Drugs

**FIGURE 9:**
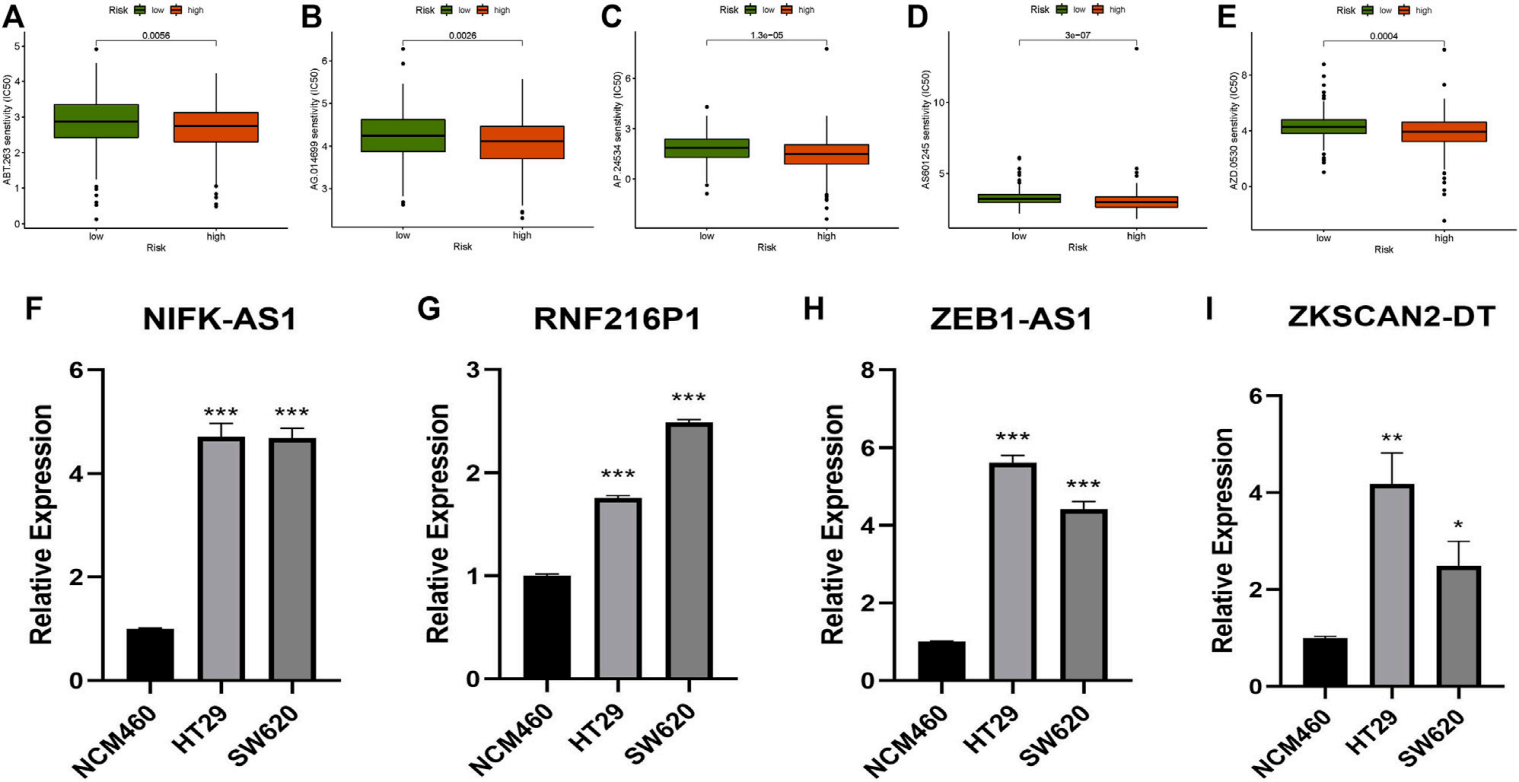
Prediction of potentially therapeutic compounds and qRT–PCR. **(A)** ABT.263, **(B)** AG.014699, **(C)** AP.24534, **(D)** AS601245, **(E)** AZD.0530, **(F–I)** Relative expression of NIFK-AS1, RNF216P1, ZEB1-AS1, and ZKSCAN2-DT in NCM460, HT29, and SW620 cell lines (Mean ± SEM). *p < 0.05, **p < 0.01, ***p < 0.001.

The sensitivity to five chemicals (AG.014699, ABT.263, AS601245, AP.24534, and AZD.0530) in the low- and high-risk groups differed considerably in the prediction of prospective chemical drugs for the treatment of colon cancer (Figures 9A–E). The sensitivity of all chemicals in the high-risk group was greater than that of the low-risk group. This discovery may aid in the treatment of CC sufferers in the future.

### Analysis of qRT–PCR

Four m7G-related lncRNAs (ZEB1-AS1, NIFK-AS1, RNF216P1, and ZKSCAN2-DT) were chosen. NCM460, HT29, and SW620 cells were used to examine these lncRNAs. The expression levels of these lncRNAs changed dramatically between tumour and normal cell lines, according to experimental evidence (Figures 9F–I). This result further validated the accuracy of our risk model.

## Discussion

Colon cancer (CC) is one of the most lethal human tumours. Over the past few years, with the progress of diagnosis and treatment technology, the total incidence of colon cancer has gradually decreased. However, the incidence of colon cancer among young patients is on the rise (Islami et al., 2021), and it is still an illness that human beings need to focus on (Biller and Schrag, 2021). Many lncRNAs have a regulatory function in the onset and progression of CC. Ren’s research demonstrated that the lncRNA RPARP-AS1 may increase the migration, proliferation and invasion of CC cells *via* the PARP-AS1/miR-125a-5p axis (Ren et al., 2021). Cen investigated the impact of lncRNA IGFL2-AS1 in colon cancer, and the findings revealed that lncRNA IGFL2-AS1 increased colon cancer cell migration, proliferation, and invasion (Cen et al., 2021). Professor Du confirmed that the lncRNA ELFN1-AS1 promotes colon cancer cell proliferation and invasion by modulating the miR-191-5p/SATB1 axis in colon cells (Du et al., 2020). Progressively increasing research has demonstrated that lncRNAs have a crucial regulatory function in CC; however, the precise mechanism is still being debated.

RNA methylation governs practically all aspects of RNA processing, and it is crucial in controlling gene expression, mRNA stability, and homeostasis. 7-Methylguanosine (m7G) RNA methylation is a recently identified type of RNA methylation that is expected to have a key function in malignancies (Zhou et al., 2021). Orellana found that METTL1-mediated tRNA modification promotes the expression of growth-promoting proteins by reshaping the “translation group” of mRNA, thus driving carcinogenic transformation (Orellana et al., 2021). After the completion of the experiment, Liu confirmed that the overexpression of METTL1 suppressed HMGA2 by upregulating let-7E miRNA, thus inhibiting the progression of CC (Liu et al., 2020). Interestingly, the methylation of RNA is intimately connected to the expression of lncRNAs, which seems to mean that m7G may affect tumorigenesis by regulating the expression of lncRNAs. However, this aspect is rarely mentioned in published studies, and up to this point, the mechanism is not totally definite. In our research, m7G-related lncRNAs were separated into distinct subgroups for the first time, and prognostic markers were constructed to comprehensively investigate the relationship between immune cell infiltration, tumour microenvironment, and m7G-related lncRNAs. We anticipate that these findings will be used to guide future clinical diagnosis and therapy of CC.

We obtained the expression data files and clinical data files for CC from the TCGA database for this study, and 29 genes associated with m7G were gathered from the published literature and GSEA website. Coexpression and univariate Cox analysis were used to identify 90 prognostic m7G-related lncRNAs. We subsequently conducted GO and KEGG analyses, which showed that m7G-related lncRNAs are mainly related to cell transcription and translation. Two subgroups were identified by consensus cluster analysis, and the survival analysis revealed disparities in survival between the two groupings. The difference between the two clusters was validated by immunological score, immune checkpoint expression level, and immune cell analysis. Cluster 2 showed a greater amount of immune checkpoint expression and a higher immunological score, and its prognosis was much better than that of Cluster 1. These findings suggested that consensus clustering is associated with patient prognosis and may be correlated which the immunological microenvironment. To forecast the prognosis of CC patients, we established a risk model with 21 signature m7G-related lncRNAs (ZKSCAN2-DT, AL512306.2, AC005014.2, U91328.1, AL354993.2, AC092944.1, LINC00997, AC004540.2, LINC02593, AC004846.1, ZEB1-AS1, AC078820.1, AC139720.2, AP003555.2, AC012313.5, AL513550.1, AC104819.3, AL137782.1, RNF216P1, AC003101.2, and AC073896.3) by LASSO regression. The risk model is remarkably dependable and related to the prognosis of CC patients, according to ROC curves, Kaplan-Meier curves, and risk curves. PCA, independent prognostic analysis, survival analysis of subgroups with differing clinicopathological characteristics, multi-index ROC curve, survival analysis of TMB, c-index curve, and model comparison again tested the reliability of our model. Immune cell/function analysis showed that m7G-related lncRNAs may be related to tumour immunity. The significant difference in the ratio of resting dendritic cells and eosinophils between the two risk groups may be helpful according to Grisaru-Tal’s and Wooster’s research. The noteworthy difference of resting dendritic cells and eosinophils in distinct risk groups may possess the potential to direct CC therapy in the future (Grisaru-Tal et al., 2020; Wooster et al., 2021). Furthermore, drug sensitivity analysis also provides a possible reference for the clinical therapy of CC, and the results of qRT–PCR confirm the trustworthiness of our research.

In published research, the lncRNA ZEB1-AS1 was considered to be tightly related to the onset and progression of many types of cancers. According to Jin’s research, the positive reciprocal looping of HIF-1/ZEB1-AS1/ZEB1/HDAC1 leads to hypoxia-induced oncogenicity and PC metastasis (Jin et al., 2021). Siena confirmed that lncRNA ZEB1-AS1 can influence the invasiveness and phenotypic transformation of melanoma through epithelial to mesenchymal transition (EMT) (Siena et al., 2019). Wu believes that ZEB1-AS1 may fuel the profitability of colorectal cancer cells by saponifying miR-141-3p (Wu et al., 2020). LncRNA LINC00997 has been discovered to perform a regulatory role in tumours in recent years. Shi’s research suggests that LINC00997 promotes colorectal cancer metastasis by targeting miR-512-3p (Shi et al., 2021). Chang believes that the LINC00997-STAT3-S100A11 axis potentially helps KIRC develop (Chang et al., 2019). These tumour-related lncRNAs are closely related to m7G. However, more m7G-related lncRNAs still lack relevant studies to confirm their relationship with tumours, and our findings may serve as a fresh reference for future studies. In our research, we firstly analysed the lncRNA related to m7G in CC by consensus clustering and established a risk model. Second, this work thoroughly investigated the association between m7G-related lncRNA prognostic markers and the tumour microenvironment, immune cell infiltration, and TMB, which may provide a new direction for future research into the predictive significance of m7G-related lncRNA markers in immunotherapy. Third, we projected several potential chemicals that may be effective for CC therapy in the future.

There are also some shortcomings in this research. First, our data source is relatively singlar, and there is a lack of more external data verification. This is mainly due to the lack of datasets with complete lncRNA sequencing data, such as TCGA databases. Second, we lack additional molecular biology experiments to verify our results. These problems are also the direction that we need to work on next.

## Conclusion

In this study, we investigated the usefulness of m7G-related lncRNAs in predicting survival, the involvement of the tumour microenvironment and immune cell infiltration, the prospective regulation mechanism of m7G-associated lncRNAs, and the prediction of suitable drugs for the treatment of CC. 21 lncRNA characteristics related with m7G may predict CC patient survival and may be beneficial for customized cancer therapy in the future.

## Data Availability

The original contributions presented in the study are included in the article/Supplementary Material, further inquiries can be directed to the corresponding authors.
