# Supplementary figures and images for: The m7G-Related Long Noncoding RNA Signature Predicts Prognosis and Indicates Tumour Immune Infiltration in Colon Cancer
Source: Front Genet. 2022 Jun 29;13:892589. doi: 10.3389/fgene.2022.892589 (PMC9277109; doi:10.3389/fgene.2022.892589)

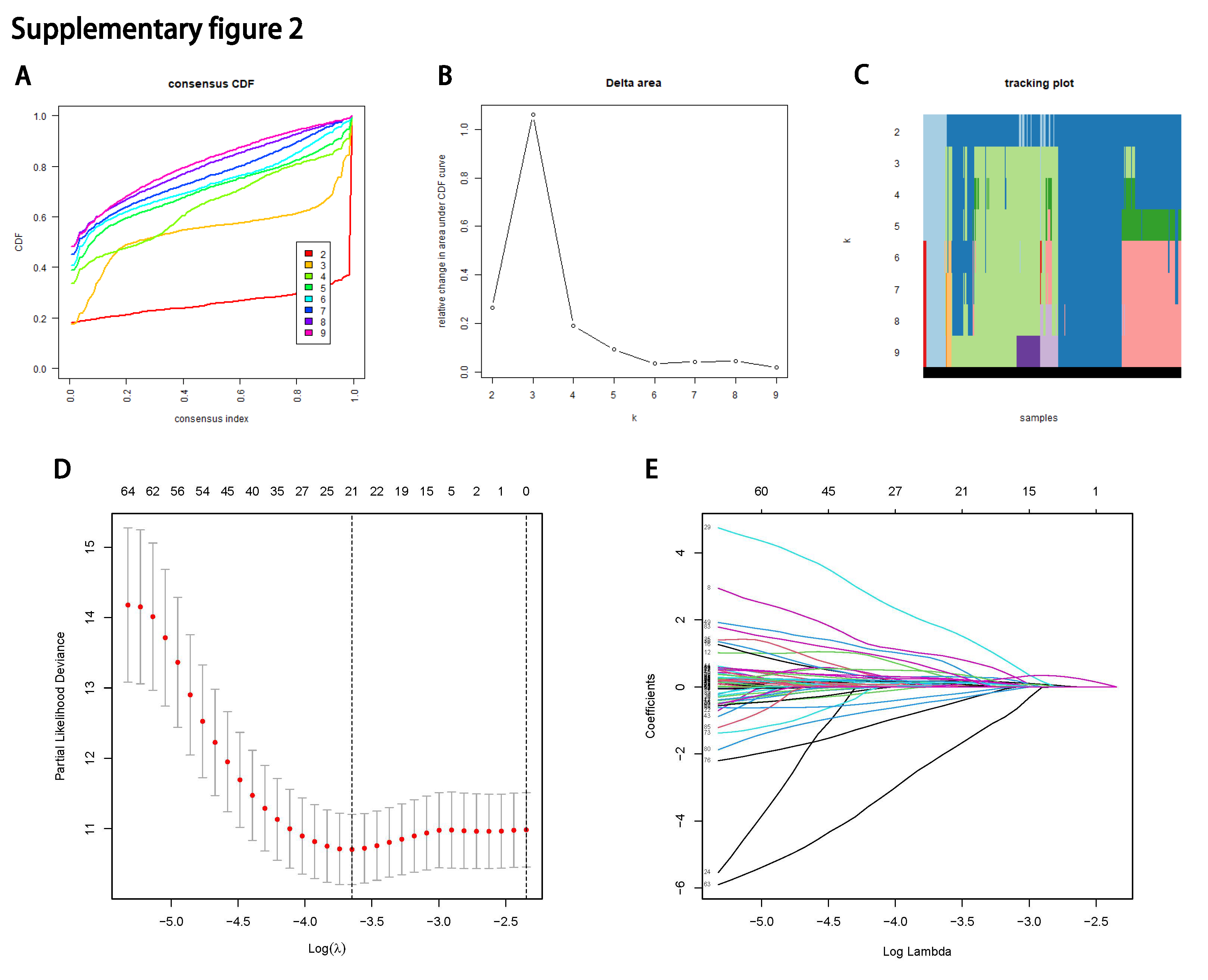

Supplement: Supplementary file 1 [file Image2.TIF]

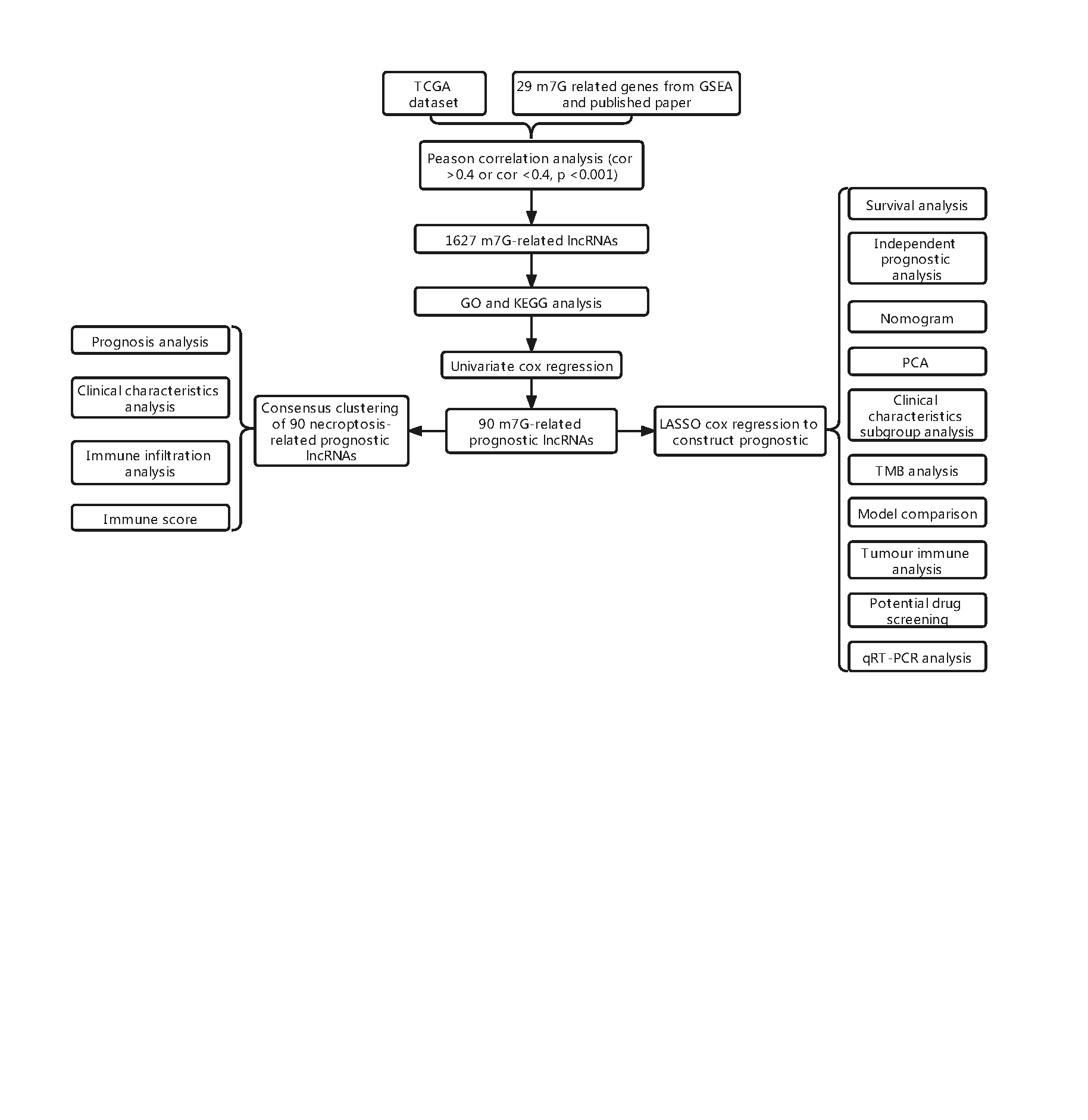

Supplement: Supplementary file 2 [file Image1.TIF]
