# Supplementary material for: The m7G-Related Long Noncoding RNA Signature Predicts Prognosis and Indicates Tumour Immune Infiltration in Colon Cancer
Source: Front Genet. 2022 Jun 29;13:892589. doi: 10.3389/fgene.2022.892589 (PMC9277109; doi:10.3389/fgene.2022.892589)
Supplement: Supplementary file 4 [file DataSheet1.DOCX]

**Table S1. m7G-related genes**

| Genes | Description |
| --- | --- |
| METTL1 | methyltransferase 1, tRNA methylguanosine |
| WDR4 | WD repeat domain 4 |
| NSUN2 | NOP2/Sun RNA methyltransferase 2 |
| DCP2 | decapping mRNA 2 |
| DCPS | decapping enzyme, scavenger |
| NUDT10 | nudix hydrolase 10 |
| NUDT11 | nudix hydrolase 11 |
| NUDT16 | nudix hydrolase 16 |
| NUDT3 | nudix hydrolase 3 |
| NUDT4 | nudix hydrolase 4 |
| NUDT4B | nudix hydrolase 4B |
| AGO2 | argonaute RISC catalytic component 2 |
| CYFIP1 | cytoplasmic FMR1 interacting protein 1 |
| EIF4E | eukaryotic translation initiation factor 4E |
| EIF4E1B | eukaryotic translation initiation factor 4E family member 1B |
| EIF4E2 | eukaryotic translation initiation factor 4E family member 2 |
| EIF4E3 | eukaryotic translation initiation factor 4E family member 3 |
| GEMIN5 | gem nuclear organelle associated protein 5 |
| LARP1 | La ribonucleoprotein 1, translational regulator |
| NCBP1 | nuclear cap binding protein subunit 1 |
| NCBP2 | nuclear cap binding protein subunit 2 |
| NCBP3 | nuclear cap binding protein subunit 3 |
| EIF3D | eukaryotic translation initiation factor 3 subunit D |
| EIF4A1 | eukaryotic translation initiation factor 4A1 |
| EIF4G3 | eukaryotic translation initiation factor 4 gamma 3 |
| IFIT5 | interferon induced protein with tetratricopeptide repeats 5 |
| LSM1 | LSM1 homolog, mRNA degradation associated |
| NCBP2L | nuclear cap binding protein subunit 2 like |
| SNUPN | snurportin 1 |

**Table S2. Prognosis related lncRNA.**

| **Gene** | **HR** | **HR.95L** | **HR.95H** | **P value** |
| --- | --- | --- | --- | --- |
| ZKSCAN2-DT | 1.353967 | 1.152349 | 1.59086 | 0.00023 |
| AL161729.4 | 1.358745 | 1.095729 | 1.684895 | 0.005225 |
| AC138207.5 | 1.342927 | 1.059019 | 1.702947 | 0.014967 |
| AL133477.1 | 0.5167 | 0.267715 | 0.99725 | 0.049048 |
| AL138921.1 | 4.713922 | 1.16979 | 18.99577 | 0.02922 |
| AC119396.1 | 1.844344 | 1.225271 | 2.776204 | 0.00335 |
| LINC01138 | 1.673956 | 1.163027 | 2.40934 | 0.005558 |
| AL512306.2 | 3.536701 | 1.720743 | 7.2691 | 0.000589 |
| PCED1B-AS1 | 1.23767 | 1.025259 | 1.494088 | 0.026444 |
| SNHG26 | 1.569233 | 1.09293 | 2.25311 | 0.014628 |
| AC068580.1 | 1.354679 | 1.050923 | 1.746232 | 0.01911 |
| AC005014.2 | 3.251671 | 1.183786 | 8.931819 | 0.022183 |
| AC008972.2 | 1.971945 | 1.073265 | 3.623122 | 0.028686 |
| AL391095.1 | 1.861246 | 1.068997 | 3.240643 | 0.028107 |
| AC024560.3 | 1.379295 | 1.007607 | 1.888092 | 0.044721 |
| LINC02257 | 1.312822 | 1.012147 | 1.702816 | 0.040272 |
| AC018653.3 | 1.234111 | 1.005595 | 1.514556 | 0.044076 |
| IGBP1-AS2 | 0.213771 | 0.047137 | 0.969469 | 0.045483 |
| U91328.1 | 2.384349 | 1.364697 | 4.16585 | 0.002272 |
| AL354993.2 | 1.216899 | 1.011467 | 1.464056 | 0.03745 |
| AL356417.2 | 2.251334 | 1.095632 | 4.626103 | 0.027208 |
| AC092118.2 | 5.078947 | 1.484233 | 17.37982 | 0.009622 |
| AC145285.2 | 4.352499 | 1.560745 | 12.13795 | 0.004943 |
| AC019205.1 | 18.57079 | 3.0724 | 112.2491 | 0.001459 |
| AC147651.1 | 1.945297 | 1.13476 | 3.334786 | 0.015534 |
| AC011462.4 | 1.389486 | 1.01406 | 1.903901 | 0.040673 |
| AL391422.4 | 1.842133 | 1.278561 | 2.654118 | 0.001042 |
| AP001619.1 | 1.77885 | 1.210567 | 2.613907 | 0.003356 |
| AC092944.1 | 4.684566 | 1.244349 | 17.63585 | 0.02242 |
| AC023024.1 | 4.121951 | 1.257803 | 13.50806 | 0.019351 |
| LINC00997 | 1.26096 | 1.003114 | 1.585084 | 0.046967 |
| AL512306.3 | 3.360268 | 1.485576 | 7.600688 | 0.00361 |
| AC004540.2 | 1.194339 | 1.068311 | 1.335236 | 0.0018 |
| LINC02550 | 2.046702 | 1.288819 | 3.250253 | 0.002404 |
| AC069281.2 | 1.333835 | 1.011746 | 1.75846 | 0.041075 |
| LINC02593 | 1.999373 | 1.428492 | 2.798399 | 5.37E-05 |
| AP003119.3 | 2.41654 | 1.234418 | 4.730704 | 0.01004 |
| DUBR | 2.277145 | 1.080581 | 4.798705 | 0.030485 |
| MIR4435-2HG | 1.290058 | 1.048067 | 1.587921 | 0.016265 |
| AC004264.1 | 1.306217 | 1.004579 | 1.698425 | 0.046145 |
| AC069222.1 | 3.272274 | 1.02441 | 10.45262 | 0.045429 |
| LINC00235 | 2.023297 | 1.010002 | 4.053193 | 0.046807 |
| AC027237.3 | 6.187696 | 1.485581 | 25.7728 | 0.012291 |
| DUXAP8 | 1.802395 | 1.023129 | 3.175191 | 0.041439 |
| AC139149.1 | 3.266709 | 1.551316 | 6.878927 | 0.001835 |
| LINC02387 | 2.634783 | 1.182984 | 5.868282 | 0.017727 |
| AC093382.1 | 3.418537 | 1.044772 | 11.1856 | 0.042115 |
| AL360181.2 | 1.177877 | 1.007303 | 1.377336 | 0.040254 |
| AC004846.1 | 6.797369 | 1.922568 | 24.03256 | 0.002935 |
| AC006042.1 | 1.070744 | 1.009493 | 1.135712 | 0.022946 |
| AC008760.1 | 1.495099 | 1.152327 | 1.939832 | 0.002469 |
| ZEB1-AS1 | 2.165995 | 1.505076 | 3.117142 | 3.17E-05 |
| AL033384.2 | 1.572332 | 1.026983 | 2.407274 | 0.037299 |
| NIFK-AS1 | 1.766226 | 1.231401 | 2.533338 | 0.001995 |
| AC078820.1 | 1.906672 | 1.094868 | 3.320399 | 0.022596 |
| AC139720.2 | 1.357414 | 1.068759 | 1.72403 | 0.012242 |
| HCG27 | 1.649873 | 1.028239 | 2.647322 | 0.03795 |
| AC004951.4 | 8.017466 | 1.309478 | 49.08807 | 0.024347 |
| FGF14-AS2 | 1.505919 | 1.141172 | 1.987248 | 0.003814 |
| MYOSLID | 5.122059 | 1.41353 | 18.56027 | 0.012889 |
| AP003555.2 | 1.375865 | 1.203499 | 1.572917 | 2.98E-06 |
| AC119403.1 | 2.020393 | 1.365177 | 2.99008 | 0.000438 |
| AC012313.5 | 0.104897 | 0.013357 | 0.823802 | 0.03201 |
| LINC00861 | 1.496225 | 1.066907 | 2.098298 | 0.019527 |
| LINC02381 | 1.235034 | 1.050746 | 1.451642 | 0.010457 |
| AC016394.1 | 1.179535 | 1.02123 | 1.362381 | 0.024725 |
| AP001469.3 | 1.545616 | 1.136658 | 2.101713 | 0.005489 |
| AC007128.1 | 1.46316 | 1.037828 | 2.062806 | 0.029868 |
| AC234582.1 | 2.025989 | 1.229863 | 3.337471 | 0.005565 |
| AL590369.1 | 3.573989 | 1.210457 | 10.55254 | 0.021126 |
| AC074117.1 | 1.229388 | 1.02228 | 1.478457 | 0.028231 |
| MALINC1 | 2.616405 | 1.099512 | 6.226015 | 0.029672 |
| LINC01679 | 3.437909 | 1.371784 | 8.615944 | 0.00843 |
| AC007541.1 | 4.7846 | 1.362183 | 16.80567 | 0.014599 |
| AL513550.1 | 1.419588 | 1.080372 | 1.865311 | 0.011908 |
| AC104819.3 | 0.25454 | 0.081723 | 0.792811 | 0.018251 |
| AP006621.2 | 1.168783 | 1.035016 | 1.319838 | 0.011905 |
| AP001628.1 | 1.242805 | 1.035135 | 1.492138 | 0.019799 |
| ATP2B1-AS1 | 9.714656 | 3.070303 | 30.73786 | 0.000109 |
| AL137782.1 | 0.391955 | 0.187424 | 0.819687 | 0.01284 |
| RNF216P1 | 1.29443 | 1.061325 | 1.578735 | 0.01085 |
| AC025171.4 | 1.825095 | 1.031684 | 3.228673 | 0.038721 |
| AC003101.2 | 2.328688 | 1.131825 | 4.791188 | 0.021655 |
| SNHG16 | 0.877115 | 0.777096 | 0.990006 | 0.033791 |
| FAM66C | 3.800764 | 1.076928 | 13.4139 | 0.037973 |
| AL096865.1 | 1.645459 | 1.026666 | 2.637213 | 0.038517 |
| AL135999.1 | 1.762706 | 1.0822 | 2.871127 | 0.022766 |
| AC073896.3 | 0.576431 | 0.353215 | 0.940709 | 0.027485 |
| LINC01011 | 1.451196 | 1.072179 | 1.964197 | 0.015899 |
| AL137186.2 | 1.742378 | 1.125504 | 2.697353 | 0.012767 |

**Table S3. LncRNA related to risk model.**

| **Gene** | **Coef** |
| --- | --- |
| ZKSCAN2-DT | 0.176944 |
| AL512306.2 | 0.933837 |
| AC005014.2 | 0.326054 |
| U91328.1 | 0.111829 |
| AL354993.2 | 0.064972 |
| AC092944.1 | 1.771428 |
| LINC00997 | 0.105833 |
| AC004540.2 | 0.041148 |
| LINC02593 | 0.309526 |
| AC004846.1 | 0.771493 |
| ZEB1-AS1 | 0.027471 |
| AC078820.1 | 0.430076 |
| AC139720.2 | 0.162469 |
| AP003555.2 | 0.225983 |
| AC012313.5 | -2.02817 |
| AL513550.1 | 0.255834 |
| AC104819.3 | -0.56887 |
| AL137782.1 | -0.40286 |
| RNF216P1 | 0.122413 |
| AC003101.2 | 0.47174 |
| AC073896.3 | -0.24717 |
